# Nerve Ultrasound Comparison Between Transthyretin Familial Amyloid Polyneuropathy and Chronic Inflammatory Demyelinating Polyneuropathy

**DOI:** 10.3389/fneur.2021.632096

**Published:** 2021-02-26

**Authors:** Kang Du, Ke Xu, Si Cheng, He Lv, Wei Zhang, Zhaoxia Wang, Yun Yuan, Lingchao Meng

**Affiliations:** ^1^Department of Neurology, Peking University First Hospital, Beijing, China; ^2^Beijing Tiantan Hospital, Capital Medical University, Beijing, China

**Keywords:** TTR-FAP, CIDP, transthyretin, polyneuropathy, ultrasonography

## Abstract

**Backgrounds:** Transthyretin familial amyloid polyneuropathy (TTR-FAP) is frequently misdiagnosed as chronic inflammatory demyelinating polyneuropathy (CIDP) because of similar phenotypes in the two diseases. This study was intended to identify the role of nerve ultrasonography in evaluating TTR-FAP and CIDP.

**Methods:** Eighteen patients with TTR-FAP, 13 patients with CIDP, and 14 healthy controls (HC) were enrolled in this study. Consecutive ultrasonography scanning was performed in six pairs of nerves of bilateral limbs with 30 sites. The cross-sectional areas (CSAs) and CSA variability data of different groups were calculated and compared.

**Results:** Both TTR-FAP and CIDP showed larger CSAs at most sites of both upper and lower limbs than in HC groups. CIDP patients had larger CSAs than TTR-FAP patients at 8/15 of these sites, especially at U1-3, Sci2 sites (*p* < 0.01). However, the CSAs at above sites were not a credible index to differentiate TTR-FAP from CIDP with a low area under the curve (<0.8). The CSA variability of median nerves was significantly higher in CIDP than in TTR-FAP and HC groups, with high sensitivity (0.692) and specificity (0.833) to differentiate CIDP from TTR-FAP. The CSA variability of ulnar nerves was not significantly different between the three groups. For the TTR-FAP group, mean CSAs at each site were not correlated with different Coutinho stages, modified polyneuropathy disability, course of sensory motor peripheral neuropathy, Neuropathy Impairment Score, or Norfolk Quality of life-diabetic neuropathy score. The mean compound muscle action potential of ulnar nerves was negatively correlated with the mean CSAs of ulnar nerves.

**Interpretation:** TTR-FAP patients had milder nerve enlargement with less variability in CSAs of median nerves than those with CIDP, suggesting that nerve ultrasound can be a potential useful auxiliary tool to help differentiate the two neuropathies.

## Introduction

Transthyretin familial amyloid neuropathy (TTR-FAP) is a multiple systemic disorder caused by *TTR* gene mutation and characterized by extracellular deposition of transthyretin-derived amyloid fibrils in peripheral and autonomic nerves and other organs. The typical phenotype of TTR-FAP is severe progressive sensory and motor neuropathy with autonomic neuropathy among adults, and most of them with cardiomyopathy. The pathology of TTR-FAP is characterized by TTR deposition with diffuse loss of nerve fibers. However, phenotypic variability and non-disease-specific symptoms or unknown family history often delay diagnosis and lead to misdiagnosis (1), including chronic inflammatory demyelinating polyneuropathy (CIDP). Some sporadic cases present with the demyelinating process in nerve conduction studies (NCSs) (2, 3), which fulfills both clinical and electrophysiologic criteria for CIDP during initial evaluation (4). Since early differentiation of TTR-FAP from CIDP is important for the treatment of either disease, several electrophysiological studies were performed for differential diagnosis. Quantitative sudomotor test was used to distinguish CIDP from TTR-FAP with good sensitivity and specificity (5).

Nerve ultrasound is a painless tool for quick evaluation of peripheral nerve morphology. Several nerve ultrasound studies showed nerve enlargement in TTR-FAP (6–8). The cross-sectional areas (CSAs) of peripheral nerves in cases of TTR-FAP are significantly larger than those of controls, most are in the proximal nerve segments (7). Nerve ultrasound patterns can facilitate the evaluation of asymptomatic carriers, presenting as larger nerve CSAs at proximal nerve sites (8). Nerve ultrasound can also serve as a useful complementary diagnostic tool for the identification of treatment-responsive inflammatory neuropathies (9–11). Sonographic nerve enlargement was present in all patients and was most prominent in proximal segments of the median nerve and brachial plexus (9, 10), including the fascicle CSAs in CIDP (12). The nerve ultrasound finding of CIDP is different from that of demyelinating diabetic sensorimotor polyneuropathy (9). However, there has been no study so far on the nerve ultrasound comparison between CIDP and TTR-FAP. In this study, more unabridged nerve sites including both upper and lower limbs were measured and the CSA variability of CIDP and TTR-FAP patients was compared.

## Materials and Methods

### Subjects

Between June 2015 and September 2020, 18 patients (16 males and 2 females) with TTR-FAP, 13 patients (3 males and 10 females) with CIDP, and 14 healthy controls (8 males and 6 females) were recruited in Peking University First Hospital. All TTR-FAP patients were diagnosed according to the diagnostic criteria (1). For the diagnosis of definite CIDP, the diagnostic criteria proposed by the Joint Task Force of the European Federation of Neurological Societies and the Peripheral Nerve Society (EFNS/PNS) were used (4). The exclusion criteria of healthy controls were: [1] skin numbness or paresthesia; [2] muscle atrophy or weakness; [3] other disorders of the peripheral nervous system; and [4] chronic diseases of other organs (e.g., heart, brain, eye, and kidney).

The mean age of TTR-FAP patients, CIDP patients, and healthy controls was 45.8 years (range 26–64 years), 40.7 years (range 15–69 years), and 40.3 years (range 26–65 years), respectively. There was no statistically significant difference in age between the three groups (*p* = 0.587).

### Clinical Neurologic Evaluation of TTR-FAP and CIDP Patients

All TTR-FAP subjects diagnosed with mutations in the *TTR* gene were inquired about their disease history and had a focused neurological examination of measurement scales performed, including Neuropathy Impairment Score (NIS), Norfolk Quality of life-diabetic neuropathy score (Norfolk QOL-DN), and modified polyneuropathy disability (m-PND). Disease severity was estimated by Coutinho staging of TTR-FAP. Nerve conduction studies (NCSs) were performed in all TTR-FAP patients according to the standard protocol using surface stimulation and recording. The motor nerve conduction velocity (MCV) and distal compound muscle action potential (CMAP) of the bilateral median, ulnar nerves of 11 patients were included in this study. All CIDP subjects were asked about their detailed disease history, and underwent neurological examination and NCSs. Sural nerve biopsy was conducted for 17/18 of TTR-FAP patients and most of the CIDP patients (9/13). Congo red staining and TTR immunohistochemical staining were performed in 17/18 and 13/18 of the TTR-FAP patients, respectively.

### Ultrasonographic Studies

All subjects underwent peripheral nerve ultrasound using the Philips Imaging System (iU Elite, Bothell, WA, USA) that measured and recorded the bilateral median, ulnar, sciatic, tibial, common peroneal, and sural nerves. To be more specific, the 17MHz high-frequency linear array probe was used for the superficial nerves, including the median nerves, ulnar nerves, common peroneal nerves, and sural nerves, and the 9 MHz linear array probe was used for the deeper nerves, including the sciatic nerves and tibial nerves.

The CSAs at the predetermined sites of each nerve were measured by tracing just inside the hyperechoic rim of the nerve. Thirty predetermined sites were measured of all the nerves (13), including [1] 10 sites that were measured in left and right median nerves (LM & RM): LM1/RM1= wrist (entrance of the carpal tunnel at the pisiform bone level); LM2/RM2= distal forearm (the nerve reached the deep flexor digitorum and started to traverse between the deep flexor digitorum and the flexor pollicis longus); LM3/RM3= proximal forearm (the clearest point before the nerve entered pronator teres); LM4/RM4= elbow (elbow socket); LM5/RM5= upper arm (from cubital fossa to the middle of armpit). [2] 10 sites of left and right ulnar nerves (LU & RU): LU1/RU1= wrist (Guyon tube: between nerve deviation and the pisiform bone and ulnar artery); LU2/RU2= distal forearm (before the ulnar nerve branches off); LU3/RU3= proximal forearm (2/3 between the wrist and elbow); LU4/RU4= elbow (at the medial epicondyle of humerus); LU5/RU5= upper arm (from cubital fossa to the middle of armpit). [3] 4 sites in left and right sciatic nerves (LSci & RSci): LSci1/RSci1= middle thigh; LSci2/RSci2= 1/3 of mid-lower part of the thigh (before sciatic nerves were divided into common peroneal nerves and tibial nerves). [4] 2 sites in left and right tibial nerves (LTib & RTib): LTib/ RTib = popliteal fossa (just after the tibial nerves were branched off by sciatic nerves). [5] 2 sites in left and right common peroneal nerves (LPc & RPc): LPc/ RPc= capitulum fibulae. [6] 2 sites of left and right sural nerves (LSural & RSural): Lsual/Rsual = lower 1/4 of the lower leg near lateral malleolus). Left sural and right sural nerves in the TTR-FAP group lacked 11 and 4 CSA data due to sural nerves biopsy, respectively. Left sural and right sural nerves in the CIDP group lacked 5 and 4 CSA data due to sural nerves biopsy, respectively.

The measured parameters were nerve CSAs and CSA variability. The CSAs were measured at these sites of each limb. The CSA variability was defined as “maximum CSA/ minimum CSA”.

### Statistical Analysis

IBM SPSS Statistics, version 26 was used for statistical analysis. The CSAs of healthy controls showed a normal distribution, while those of CIDP and TTR-FAP showed an abnormal distribution (as evaluated by single sample K-S test). Thus, Mann-Whitney *U* test was used for evaluating differences in CSAs between TTR-FAP, CIDP and healthy control groups, as well as CSA variability between TTR-FAP and CIDP groups. Receiver operating characteristic (ROC) curve analysis was performed to evaluate the applicability of CSA variability measurements to differentiation of TTR-FAP from CIDP. The area under the curve (AUC) was calculated. The value of Youden index at its maximum was taken as the cut point for the diagnosis of TTR-FAP, and the sensitivity and specificity were calculated. Two-sided p values were calculated for all analyses; *p* < 0.05 was considered significant. Spearman analysis was used to test the correlation between CSAs and measuring scales, electrophysiological data.

### Data Availability

Anonymized data will be shared by request from any qualified investigator.

## Results

### Clinical Data of TTR-FAP and CIDP

Of the 18 TTR-FAP patients, 9 initially developed limb paresthesia, followed by other onset symptoms such as alternating diarrhea and constipation (ADC) in three patients, sexual dysfunction in three patients, blurred vision in two patients and constipation in one patient. All these patients presented with sensorimotor peripheral neuropathy and autonomic neuropathy, 12 suffered from asymptomatic cardiac hypertrophy, and 5 developed vitreous opacity. *TTR* gene screening was performed, with Val30Met mutation in three patients, Ala97Ser, Glu42Gly, Gly47Arg, and Lys35Asn mutation in two patients, respectively, Ala36Pro, Phe33Leu, Phe33Val, Gly83Arg, Ser77Phe, Ser77Tyr, and Val28Ser mutation in one patient, respectively. NCSs examination was also performed in all these patients: 12 presented with axonal impairment, and 6 with a mixed neuropathy. In clinical staging, 10 of these patients were divided into Coutinho stage I, and the remaining into Coutinho stage II or III.

For CIDP patients, proximal and/or distal limbs weakness were manifested, with or without paresthesia. The mean course of disease was 3.4 ± 2.2 years. Laboratory examination of cerebrospinal fluid was conducted in 8/13 of these patients with cytoalbuminologic dissociation. All the patients undergoing NCSs examination accorded with the presentation of demyelination. All patients received immunotherapy that turned out to be partly or completely effective.

Pathologically, positive Congo red staining in sural nerve biopsy was seen in 10/17 of TTR-FAP patients, positive TTR immunohistochemistry in 6/13 of these patients. All patients with nerve biopsy pathologically presented with axonal neuropathy with moderate to severe loss of both large and small myelinated nerve fibers as well as unmyelinated nerve fibers. Of the 9 CIDP patients who had sural nerve biopsy performed, variation in the density of myelinated fibers among fascicles was observed in 5 patients, infiltration of macrophage in 5 patients, and thin myelin sheath or onion-bulb formation in 6 patients. All these patients had mild to moderate loss of myelinated fibers, especially large-diameter ones.

### Ultrasonographic Findings

#### Comparison of CSAs Between TTR-FAP, CIDP, and HC

**Figure 1:**
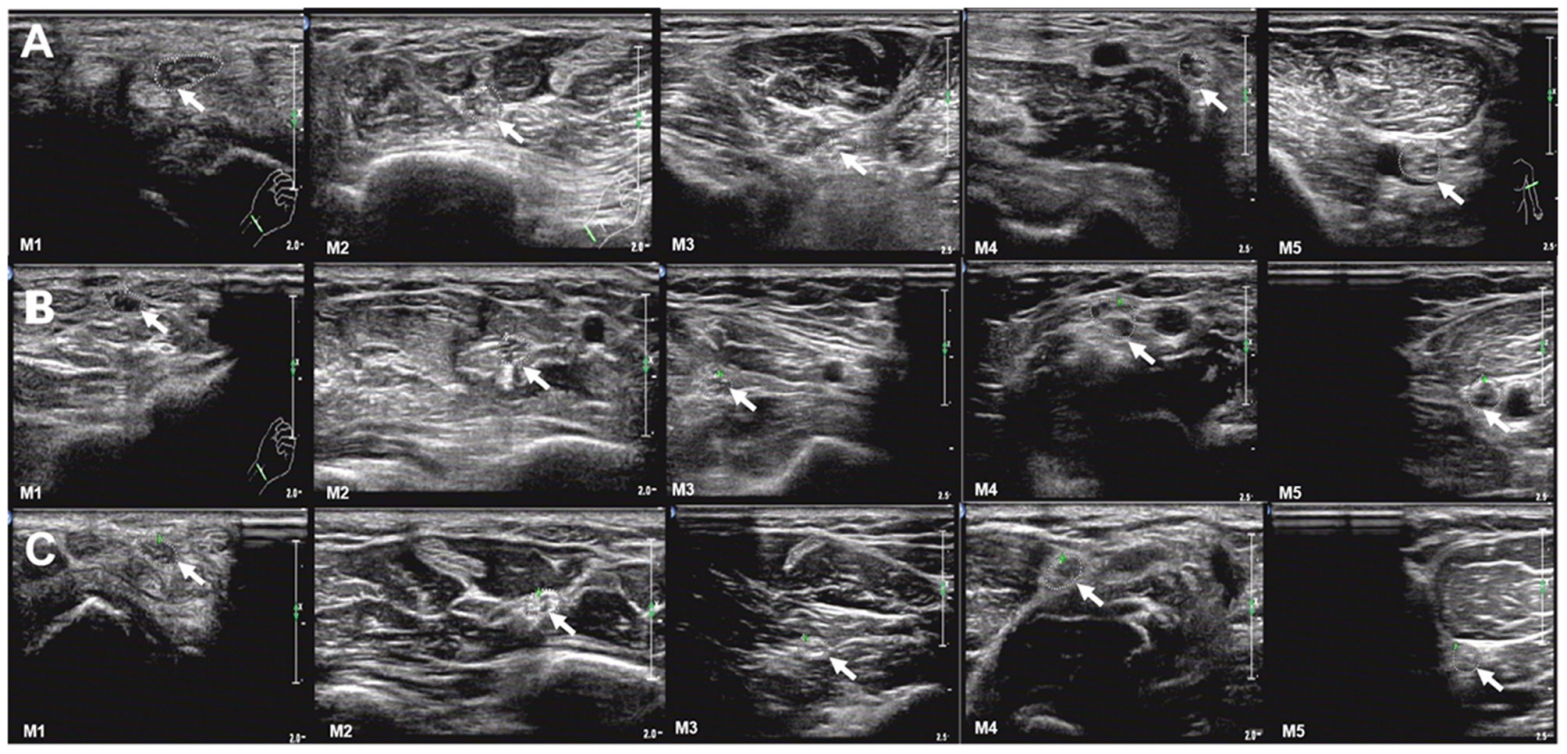
Examples of ultrasound cross-sections showing measurements of cross-sectional area of median nerves in M1-5 between the three groups. **(A)** Homogeneous enlargement was observed at sites M1-5 of a TTR-FAP patient. **(B)** Segmental enlargement was observed at sites M1-5 of median nerve in a CIDP patient. **(C)** The normal CSAs at sites M1-5 of a healthy control.

**Figure 2:**
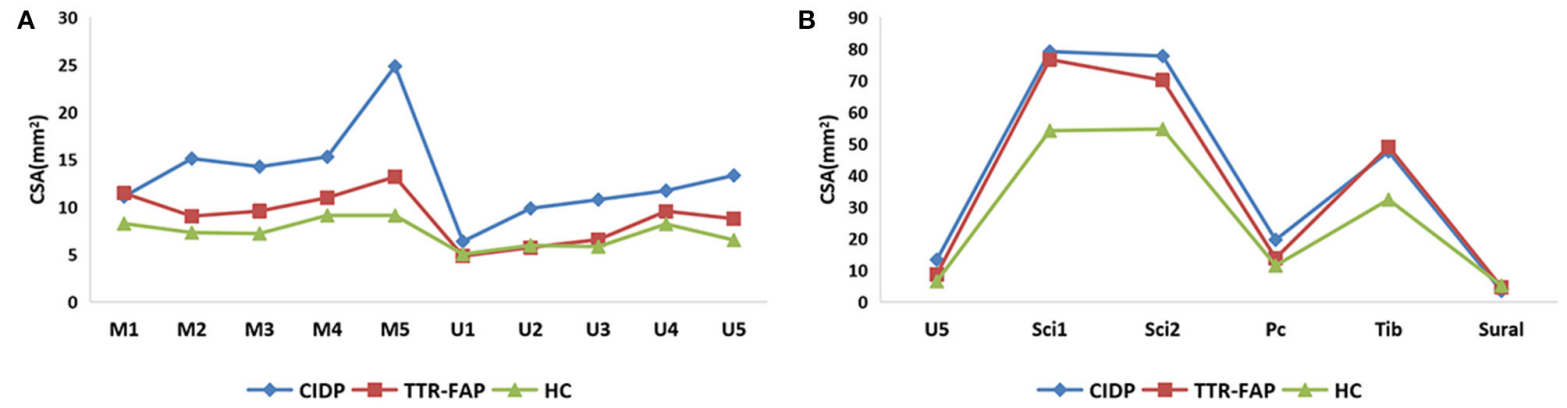
A general view of mean CSAs at sites of upper limbs **(A)** and lower limbs **(B)** between the three groups. **(A)** Clearly presented the CSAs in upper limbs, and revealed most CSAs of TTR-FAP groups at bilateral median nerves were higher than those of HC groups but lower than those of CIDP groups. **(B)** Showed most CSAs of TTR-FAP groups were between those of CIDP and HC groups in lower limbs.

The mean CSAs at 15 different sites of all nerves in each group were shown in Table 1. The mean CSAs values in the TTR-FAP group were statistically higher than those of the HC group at most sites of median, sciatic, tibial nerves, especially in median nerves and sciatic nerves (all *p* < 0.05), including M1-M3, M5, U5, Sci1, Sci2, Pc, Tib sites. The CSAs at most sites of ulnar nerves were not higher than those of the HC groups, except U5 site, which was the proximal site of ulnar nerves. The CSAs of proximal sites of median nerves (M5) and sciatic nerves (Sci1) in the TTR-FAP group were also significantly higher than in the HC group (Figures 1, 2).

**Table 1:** Comparison of CSAs at different nerve sites of upper and lower limbs in TTR-FAP, CIDP and healthy controls (mm^2^).

**Sites**	**Mean CSAs (mm^**2**^) of HC**	**Mean CSAs (mm^**2**^) of TTR-FAP**	**Mean CSAs (mm^**2**^) of CIDP**	***P* value (HC vs. TTR-FAP)**	***P* value (TTR-FAP vs. CIDP)**	***P* value (HC vs. CIDP)**
M1	8.26 (1.65)	11.46 (3.19)	11.1 (3.83)	0.000^**^	0.628	0.001^**^
***M2***	7.31 (1.36)	9.04 (2.11)	15.11 (10.07)	0.001^**^	0.029^*^	0.000^**^
M3	7.21 (1.69)	9.57 (2.63)	14.26 (10.68)	0.000^**^	0.180	0.000^**^
***M4***	9.12 (1.97)	10.98 (3.07)	15.29 (8.32)	0.065	0.015^*^	0.000^**^
M5	9.11 (1.92)	13.19 (3.82)	24.84 (18.45)	0.000^**^	0.058	0.000^**^
***U1***	5.04 (1.74)	4.84 (1.56)	6.37 (2.67)	0.456	0.004^**^	0.049^*^
***U2***	5.95 (1.26)	5.74 (1.72)	9.86 (5.02)	0.267	0.000^**^	0.005^**^
***U3***	5.83 (1.39)	6.56 (1.97)	10.78 (6.92)	0.112	0.001^**^	0.000^**^
U4	8.21 (1.97)	9.56 (4.07)	12.2 (8.52)	0.583	0.284	0.073
***U5***	6.52 (2.01)	8.77 (3.34)	15.76 (15.25)	0.003^**^	0.049^*^	0.000^**^
Sci1	54.15 (15.7)	76.68 (19.59)	82.36 (39.19)	0.000^**^	0.936	0.002^**^
***Sci2***	54.67 (14.49)	70.09 (20.31)	118.92 (70.48)	0.001^**^	0.005^**^	0.000^**^
***Pc***	11.49 (3.79)	13.83 (3.65)	19.63 (11.93)	0.024^*^	0.022^*^	0.001^**^
Tib	32.36 (7.58)	49.09 (12.63)	47.57 (17.08)	0.000^**^	0.476	0.000^**^
Sural	5.16 (1.29)	4.62 (1.59)	5.31 (1.42)	0.075	0.064	0.590

*Mean (SD)*.

* *Significance at 0.05 level*.

** *Significance at 0.01 level*.

*All significant p values are printed in bold, and discriminative sites of TTR-FAP and CIDP groups in italics plus bold*.

*CSAs, cross-sectional areas; CIDP, chronic inflammatory demyelinating polyneuropathy; TTR-FAP, transthyretin familial amyloid polyneuropathy; HC, healthy control*.

*M, median nerve; U, ulnar nerve; Sci, sciatic nerve; Tib, tibial nerve; Pc, common peroneal nerve; Sural, sural nerve*.

The mean CSAs values at 8 sites of the TTR-FAP group were lower than in CIDP with significant difference, including M2, M4, U1-3, U5, Sci2, Pc sites. For CIDP groups, the CSAs at all sites were higher in HC groups intuitively, but were not significantly different at two sites (U4 and Sural) (Figures 1, 2).

#### Comparison of CSA Variability Between TTR-FAP and CIDP

Furthermore, the CSA variability of median nerves and ulnar nerves between the three groups was calculated. It was found that CSA variability of median nerves in CIDP groups was significantly higher than in TTR-FAP and HC groups, but there was no significant difference between TTR-FAP and HC groups. For ulnar nerves, the CSA variability between the three groups was not significantly different (Table 2).

**Table 2:** Comparison of CSA variability of median/ulnar nerve between disease groups and control group.

	**TTR-FAP**	**CIDP**	**HC**	***P*** **value**		
				**TTR-FAP v.s. CIDP**	**TTR-FAP v.s. HC**	**CIDP v.s. HC**
M-CSA-V	1.58 (0.32)	3.06 (1.88)	1.59 (0.44)	0.000^**^	0.074	0.000^**^
U-CSA-V	2.14 (0.47)	3.22 (2.50)	1.95 (0.54)	0.608	0.823	0.057

*Mean (SD)*.

** *Significant difference at 0.01 level*.

*M/U-CSA-V: CSA variability of median/ulnar nerve, defined as “maximum CSA/ minimum CSA”. All significant p values are printed in bold*.

#### The ROC of CSA and CSA Variability for Differentiating Between TTR-FAP and CIDP

**Figure 3:**
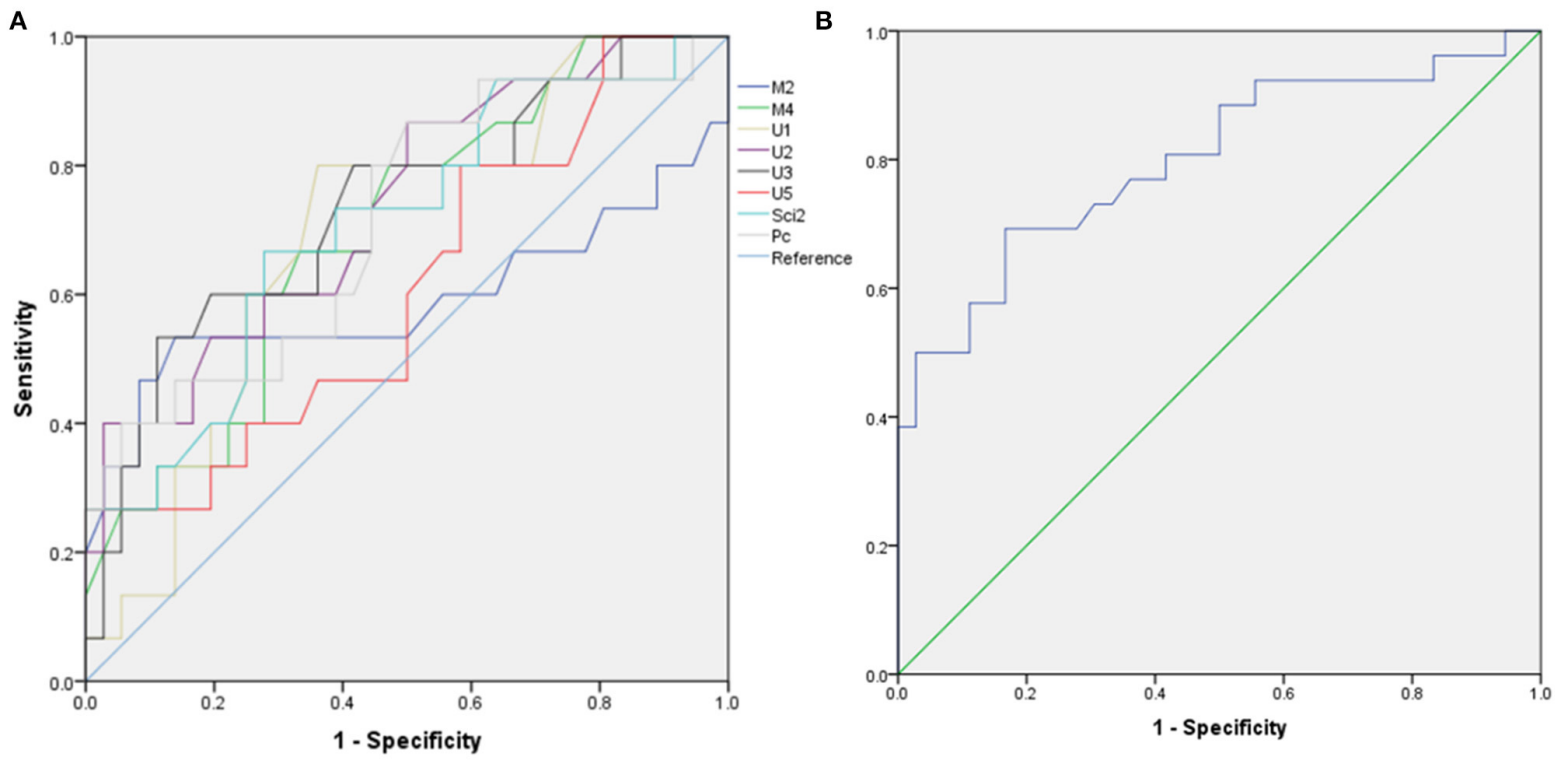
Receiver operating characteristic (ROC) curve for differentiating transthyretin familial amyloid polyneuropathy (TTR-FAP) from chronic inflammatory demyelinating polyneuropathy (CIDP). **(A)** Mean CSAs at M2, M4, U1-3, U5, Sci2, Pc sites between the two groups. **(B)** CSA-variability of median nerves between the two groups.

Based on the results observed in Tables 1, 2, we went to performed the ROC curve of CSAs and CSA variability for differentiating between TTR-FAP and CIDP. Figure 3 showed the ROC curve analyses of the mean CSAs of the discriminative sites in each nerve and CSA variability in median nerves. AUC and cutoff values were shown in Table 3. There was no significant difference between the two groups in CSAs at M2 and U5 sites (*p* > 0.05), except M4, U1-3, U5, Sci2, Pc sites. However, the AUC above was not high (all AUC <0.8). For CSA variability in median nerves, the AUC was 0.8 with high sensitivity (0.692) and specificity (0.833).

**Table 3:** The AUC, suggested cutoff values, sensitivity, and specificity of CSA and CSA variability in differentiating between TTR-FAP and CIDP.

	**AUC**	**Cutoff values**	**Sensitivity**	**Specificity**	***p* value**
M-CSA-V	0.8	1.77	0.692	0.833	0.000^**^
M2	0.59	11.95	0.533	0.861	0.316
M4	0.699	10.75	0.667	0.667	0.026^*^
U1	0.695	4.45	0.8	0.639	0.029^*^
U2	0.736	9.2	0.4	0.972	0.008^**^
U3	0.734	8.2	0.533	0.889	0.009^**^
U5	0.61	19.45	0.267	1	0.219
Sci2	0.707	78.8	0.667	0.722	0.021^*^
Pc	0.715	13.15	0.867	0.5	0.016^*^

** *Significant difference at 0.01 level*.

* *Significant difference at 0.05 level*.

*AUC, area under the curve; M-CSA-V, CSA-Variability of median nerve. All significant p values are printed in bold*.

#### Correlation of CSAs With Electrophysiological Data and Clinical Measurement Scales of TTR-FAP

All the measured sites between Coutinho stage I (*n* = 10) and Coutinho stage II/III (*n* = 8) of TTR-FAP patients were compared. However, the CSAs of these two groups at each site were not significantly different (all *p* > 0.05) (Table 4).

**Table 4:** Comparison of CSAs in different Coutinho stages of each measurement site in TTR-FAP group.

**Sites**	**Mean CSA of Stage I (mm^**2**^)**	**Mean CSA of Stage II/III (mm^**2**^)**	***P* value**
M1	11.54 (3.23)	11.36 (3.25)	0.69
M2	9 (2.16)	9.09 (2.1)	0.949
M3	9.52 (3.02)	9.64 (2.14)	0.774
M4	11.19 (3.48)	10.73 (2.54)	0.987
M5	13.21 (3.7)	13.16 (4.09)	0.691
U1	4.38 (0.84)	5.41 (2.04)	0.299
U2	5.38 (1.38)	6.18 (2.02)	0.134
U3	6.23 (1.9)	6.98 (2.04)	0.082
U4	9.23 (3.81)	9.98 (4.46)	0.474
U5	8.34 (3.56)	9.3 (3.06)	0.119
Sci1	78.51 (22.7)	74.4 (15.25)	0.924
Sci2	70.93 (24.28)	69.05 (14.6)	0.524
Pc	14.35 (3.53)	13.18 (3.8)	0.339
Tib	48.24 (12.8)	50.15 (12.75)	0.373
Sural	4.27 (1.03)	5.1 (2.1)	0.455

*Mean (SD)*.

**Figure 4:**
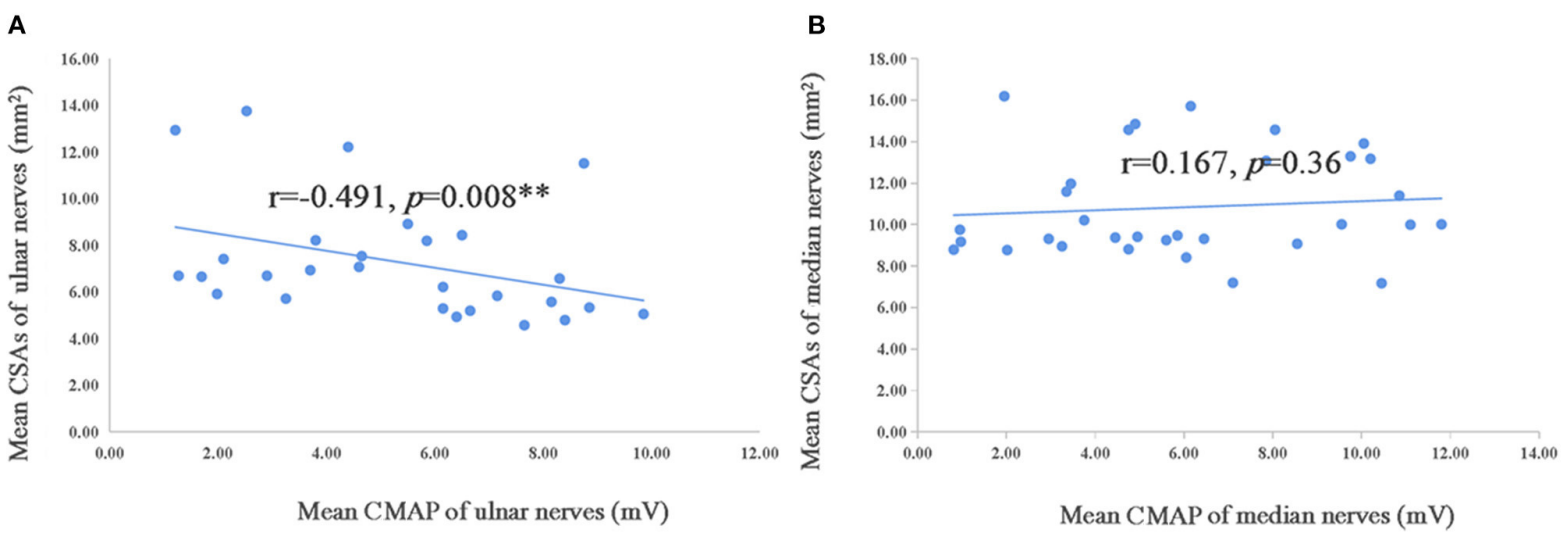
Correlation of mean CSAs and CMAP of ulnar and median nerves in TTR-FAP group. The Spearman correlation coefficient was calculated. **Significance at 0.01 level. CMAP, compound motor action potential.

In addition, the correlation analysis was conducted of mean CSAs in median and ulnar nerves and of Neuropathy Impairment Score (NIS) that included one item for reflection of muscle weakness, modified polyneuropathy disability (m-PND), Norfolk Quality of life-diabetic neuropathy score (Norfolk QOL-DN), the course of sensory motor peripheral neuropathy (SMPN) and electrophysiological data in TTR-FAP patients. However, no correlation was observation between all these indexes (Table 5), except the mean CMAP of ulnar nerves, which was negatively correlated with mean CSAs with statistically significant difference (*r* = −0.491, *p* = 0.008) (Figure 4).

**Table 5:** Correlation analysis between CSAs at different sites and clinical as well as electrophysiological data of TTR-FAP patients.

	**Mean CSAs of median nerves**	**Mean CSAs of ulnar nerves**	**Mean CSAs of sciatic nerves**	**Mean CSAs of common peroneal nerves**	**Mean CSAs of tibial nerves**	**Mean CSAs of sural nerves**	**Mean CSAs at M-5 sites**	**Mean CSAs at Sci-1 sites**
MCV of median nerves	*r* = 0.133, *p* = 0.469	NA	NA	NA	NA	NA	*r* = 0.097, *p* = 0.596	NA
Mean CMAP of median nerves	*r* = 0.167, *p* = 0.36	NA	NA	NA	NA	NA	*r* = 0.177, *p* = 0.331	NA
MCV of ulnar nerves	NA	*r* = −0.095, *p* = 0.645	NA	NA	NA	NA	NA	NA
Mean CMAP of ulnar nerves	NA	*r* = −0.491, ***p*** **=** 0.008^**^	NA	NA	NA	NA	NA	NA
MCV of common peroneal nerves	NA	NA	NA	*r* = −0.255, *p* = 0.326	NA	NA	NA	NA
Mean CMAP of common peroneal nerves	NA	NA	NA	*r* = −0.34, *p* = 0.096	NA	NA	NA	NA
MCV of tibial nerves	NA	NA	NA	NA	*r* = −0.457, *p* = 0.056	NA	NA	NA
Mean CMAP of tibial nerves	NA	NA	NA	NA	*r* = −0.126, *p* = 0.565	NA	NA	NA
Course of SMPN	*r* = −0.015, *p* = 0.954	*r* = 0.351, *p* = 0.153	*r* = 0.052, *p* = 0.838	*r* = 0.012, *p* = 0.961	*r* = 0.259, *p* = 0.299	*r* = 0.035, *p* = 0.895	*r* = −0.026, *p* = 0.919	*r* = 0.109, *p* = 0.668
NIS	*r* = 0.026, *p* = 0.919	*r* = 0.408, *p* = *0.093*	*r* = 0.071, *p* = 0.779	*r* = −0.058, *p* = 0.82	*r* = 0.337, *p* = 0.172	*r* = −0.079, *p* = 0.764	*r* = −0.043, *p* = 0.864	*r* = 0.072, *p* = 0.776
m-PND	*r* = 0.092, *p* = 0.717	*r* = 0.463, *p* = *0.053*	*r* = 0.115, *p* = 0.651	*r* = 0.005, *p* = 0.984	*r* = 0.148, *p* = 0.559	*r* = 0.037, *p* = 0.888	*r* = 0.057, *p* = 0.823	*r* = 0.125, *p* = 0.622
Norfolk QOL-DN	*r* = −0.045, *p* = 0.861	*r* = 0.396, *p* = 0.104	*r* = −0.012, *p* = 0.962	*r* = −0.17, *p* = 0.501	*r* = 0.01, *p* = 0.969	*r* = 0.159, *p* = 0.542	*r* = −0.098, *p* = 0.699	*r* = −0.01, *p* = 0.969
NIS-muscle weakness	*r* = 0.02, *p* = 0.938	*r* = 0.324, *p* = 0.19	*r* = 0.009, *p* = 0.971	*r* = −0.091, *p* = 0.719	*r* = 0.302, *p* = 0.223	*r* = −0.077, *p* = 0.769	*r* = −0.078, *p* = 0.759	*r* = 0.004, *p* = 0.987

*Spearman analysis was used to test the correlation between CSAs and electrophysiology, as well as measuring scales*.

** *Significant correlation at 0.01 level*.

*All significant p values are printed in bold*.

*NA, not available; M-5, site-5 of bilateral median nerves; Sci, site-1 of bilateral sciatic nerves; SMPN, sensory motor peripheral neuropathy; NIS, Neuropathy Impairment Score; NIS-muscle weakness, Neuropathy Impairement Score only including the items of muscle weakness; m-PND, modified polyneuropathy disability; Norfolk QOL-DN, Norfolk Quality of life-diabetic neuropathy score*.

## Discussion

The clinical symptoms and electrophysiology might be similar in TTR-FAP and CIDP patients. In TTR-FAP, destruction of myelin due to amyloid deposition might be related to nerve conduction abnormalities mimicking CIDP (5). Initial electrodiagnostic conclusions of CIDP were confirmed in only 45% of misdiagnosed studies (14).

Our study conformed with the findings of previous studies (6, 7, 15, 16) that thickness of peripheral nerves existed in TTR-FAP patients. The mean CSAs of TTR-FAP patients were higher than those of healthy controls at most sites. Enlargement of peripheral nerves has been reported in previous studies (6, 7). The CSAs at proximal sites of measurable nerves (median nerves, ulnar nerves and sciatic nerves) were significantly higher than those of healthy controls, compared with distal sites in a same nerve. All this was compatible with the findings of previous studies for nerve ultrasound and magnetic resonance neurography in TTR-FAP patients (7, 8, 15). Moreover, prominent enlargement of peripheral nerves at proximal sites was not common in most axonal neuropathies (17, 18), which could help distinguish TTR-FAP from other axonal neuropathies. For note, distal enlargement of median nerves was also observed in our study, which might associate with carpal tunnel syndrome in these TTR-FAP patients (8).

To our knowledge, there were no studies on how nerve ultrasound was used for comparing CIDP with TTR-FAP. A recent case report on TTR-FAP said that the CSAs were not different between CIDP patients and those with TTR-FAP. Instead, nerve ultrasound features of TTR-FAP could increase the incidence of misdiagnosis of CIDP. However, only one TTR-FAP patient was involved in this case (16). We found different results of CSAs between the two groups, and revealed that CSAs of enlarged peripheral nerves of TTR-FAP patients were lower than those of CIDP patients at 8/15 of sites with significant difference, especially at sites of U1-3, Sci2 (Table 1). A second point that had been neglected by other studies was the fluctuation of different CSAs in a same nerve (i.e., median and ulnar nerves) among patients with CIDP (19), which might be an auxiliary index for differentiating CIDP and TTR-FAP. The CSAs of median nerves in TTR-FAP patients were not all significantly higher than in CIDP patients, but CSA variability of the median nerves might help to differentiate CIDP from TTR-FAP due to its relatively high sensitivity and specificity. It was also speculated that the CSAs of ulnar nerves might be a potentially useful indicator for differentiating CIDP from TTR-FAP, unlike the CSAs variability of ulnar nerves.

The nerve ultrasound results may be based on pathological changes. The TTR-FAP was an axonal neuropathy and the loss of nerve fibers was diffuse and regular (20). The possible pathophysiological mechanisms have been clarified as amyloid deposits-vulnerable to compression-compression sites edema, fibrosis, thickened endoneurium, perineurium and the small vessel walls, as well as nerve fiber degeneration (7, 21). CIDP was a demyelination neuropathy characterized by infiltration of macrophage and variation in myelinated fiber density among fascicles due to focal myelinated fiber loss or onion-bulb formation (22, 23).

Previous studies suggested that disease duration, stage of TTR-FAP, or PND stage were not correlated with CSAs of median nerves (7), which was why we evaluated ulnar nerves more comprehensively. Similarly, we confirmed that disease severity, including m-PND and Coutinho staging, was not associated with CSAs in TTR-FAP patients in our study. The correlation between NIS, Norfolk QOL-DN and mean CSAs of each nerve was not observed.

Interestingly, the mean CMAP of ulnar nerves was negatively correlated with the mean CSAs in our study, suggesting that the CSAs of ulnar nerves might be used to monitor the disease severity, but further studies are needed. The negative correlation between CMAP and CSAs was observed in median nerves in previous studies (7), but not in our current study.

This study had several limitations. The sample size of TTR-FAP patients with electrophysiological data and CIDP patients was not big enough, so more subjects registered will be needed in the future. CIDP itself is a heterogeneous disease, so the CSA of each site may be affected by different disease subtypes and activities. Compared with pathological examination and genetic testing, which are the golden standard to differentiate TTR-FAP and CIDP, nerve ultrasound can only be considered as an auxiliary tool, with the non-invasive and convenient advantages.

In conclusion, our study showed TTR-FAP patients had milder nerve enlargement with less variability in CSAs of median nerves than their CIDP counterparts, suggesting that nerve ultrasound is a potential useful auxiliary tool in differentiating the two neuropathies.

## Data Availability Statement

The raw data supporting the conclusions of this article will be made available by the authors, without undue reservation.

## Ethics Statement

The studies involving human participants were reviewed and approved by the clinical research ethics committee of Peking University First Hospital. The patients/participants provided written informed consent to participate in this study.

## Author Contributions

KD: acquisition of data, completion of statistical analysis, and drafting of the initial manuscript and writing of the final manuscript. KX and SC: ultrasonography, study concept and design, and critical revision of the manuscript. HL, WZ, and YY: study concept and design, and critical revision of the manuscript. LM: data review, interpretation of results, and revision of the initial draft. All authors contributed to the article and approved the submitted version.

## Conflict of Interest

The authors declare that the research was conducted in the absence of any commercial or financial relationships that could be construed as a potential conflict of interest.
